# The Effect of Combined Ultrasonic Tip and Mechanized Instrumentation on the Reduction of the Percentage of Non-Instrumented Surfaces in Oval/Flat Root Canals: A Systematic Review and Meta-Analysis

**DOI:** 10.7759/cureus.50041

**Published:** 2023-12-06

**Authors:** Marcella Dewes Cassal, Pedro Cardoso Soares, Marcelo dos Santos

**Affiliations:** 1 Department of Restorative Dentistry, Faculty of Dentistry, University of São Paulo, São Paulo, BRA

**Keywords:** ultrasonic tip, root canal preparation, flattened root canals, oval root canals, micro-computed tomography, endodontics

## Abstract

This systematic review and meta-analysis aimed to evaluate the effectiveness of the ultrasonic tip associated with mechanized instrumentation in reducing the percentage of non-instrumented surfaces of human teeth with oval or flattened root canals. The study followed the Preferred Reporting Items for Systematic Review and Meta-Analysis (PRISMA) guidelines. Pubmed/MEDLINE (Medical Literature Analysis and Retrieval System Online), Scopus, Embase, Web of Science, and Cochrane Library were searched for literature published till October 2022. Only in vitrostudies were included, which compared conventional mechanized endodontic treatment alone against conventional endodontic treatment in association with ultrasonic tips, evaluating the reduction in the percentage of non-instrumented walls (computed microtomography). Four studies were eligible for qualitative and quantitative analysis. The majority of the studies scored low for risk of bias. The meta-analysis comparing protocols demonstrated a reduced percentage of non-instrumented walls in groups using ultrasonic tips after the conventional mechanized technique (p<0.01) with a confidence interval of 1.04 (95%CI: 0.59, 1.50). The ultrasonic tip associated with mechanized instrumentation demonstrates a significant reduction in the percentage of non-instrumented surfaces in oval or flattened canals.

## Introduction and background

The enlargement, shaping, cleaning, and decontamination of the root canal system (RCS) are objectives of endodontic therapy and are obtained through the mechanical action of endodontic instruments combined with the irrigant action [1,2]. Rotating and reciprocating nickel-titanium (NiTi) systems are widely used to model and shape root canals. However, regardless of the type of kinematics, considerable percentages of non-instrumented areas are observed after the conventional mechanized endodontic treatment in studies that evaluated the ability to clean instruments [3-9].

This limitation is related to the anatomical complexity of the oval or flat root canal system as they have a transverse configuration with a bucco-lingual diameter two to four times greater than the mesio-lingual diameter [10]. Furthermore, most endodontic instruments available on the market have a circular morphology and operate in a centralized position of oval root canals, characteristics that might lead to uninstrumented areas [2,11].

These uninstrumented regions can harbor remains of necrotic tissue and residual biofilm, in addition to residues arising from the cutting action of instruments on the dentin. This organic and inorganic debris, also called dentinal magma, can be pushed into the buccal and lingual recessions during preparation, possibly remaining in these flat areas, walls, and irregularities [2,11]. Such an event can prevent auxiliary chemical substances from penetrating these regions, compromising the effectiveness of cleaning, especially in infected channels, and can serve as a potential source of persistent infection [12-15].

Since the first described ultrasound use by Richman in 1957, it became commonly used in all stages of this endodontic therapy. The main objective of ultrasonic tip use is to optimize mechanical debridement, increasing the number of surfaces touched and promoting uniform preparation around the perimeter of the canal. The ultrasonic tips are a safe instrument, having a relatively low cost. Their thin and long design allows access to difficult and irregular areas of the root canals [16].

Various methodologies have been used to analyze in detail the results of the action of instruments [17]. Micro-computed tomography (micro-CT) is a widely used technology as it is a non-destructive method that allows the acquisition of three-dimensional models of internal structures, allowing the evaluation of the reduction of non-instrumented areas of root canals compared to the original anatomy that is observed before the intervention of endodontic procedures [9,11].

Although there are studies in the literature on the use of the ultrasonic tip associated with mechanical preparation with NiTi files, to date there is no systematic review and meta-analysis that analyzes the reduction percentage of non-instrumented areas between the techniques. For this reason, we evaluated in vitro studies that compared groups that performed mechanical preparation with or without the use of ultrasonic tips on oval or flat root canals analyzed by micro-CT.

## Review

Material and methods

This systematic review followed the Preferred Reporting Items for Systematic Review and Meta-Analysis (PRISMA) guidelines and the Cochrane Handbook [18,19]. The project is registered in the International Prospective Register of Systematic Review (PROSPERO) (number CRD42023409389).

Research Question

The eligibility criteria followed the PICO (population, intervention, comparison, and outcome) strategy, in which population (P): extracted permanent human teeth with oval or flattened root canals, Intervention (I): the use of an ultrasonic tip to complement root canal debridement, Comparison (C): the use of mechanized instruments in the preparation of root canals, and outcome (O): the reduction in the percentage of non-instrumented surfaces. Thus, the research question was “Does ultrasonic tip usage in association with mechanized instrumentation reduce the percentage of non-instrumented surfaces after the preparation of oval or flat root canals?”

Eligibility Criteria

The inclusion criteria were: in vitro laboratory studies, studies that used human teeth with oval or flattened roots with complete rhizogenesis, without root caries, without root resorption, without fracture, and previous endodontic treatment, studies that evaluated the percentage of non-instrumented surfaces before and after using the ultrasonic tip as a supplementary protocol for root canal preparation, and studies that used micro-CT as an evaluation tool in techniques that combined the use of an ultrasonic tip associated with mechanized preparation. There were no language or year of study restrictions. Literature reviews, systematic reviews, randomized and non-randomized clinical trials, clinical case reports, articles without available full text, and studies with animal models were excluded.

Search Strategy

Two examiners (MDC and PCS) performed the search procedures independently in the following electronic databases: PubMed/MEDLINE (Medical Literature Analysis and Retrieval System Online), Web of Science, Embase, Scopus, and Cochrane Library, for articles published until October 2022. The search strategy development began with PubMed/MEDLINE, and later, the authors adapted the search for each database. Search terms included “ultrasonic tip,” “ultrasonic therapy,” “root canal preparation,” “root canal therapy,” “X-ray microtomography,” and “Micro-CT.”

Review Methods

After searching the databases, the two reviewers selected titles and abstracts independently. If the studies met the inclusion criteria, or in cases of missing information for the decision, the reviewers performed an analysis of the full text. Additionally, to ensure the level of agreement between reviewers before the inclusion and exclusion phase, the reviewers used 10% of the sample to conduct Cohen Kappa. Studies inclusion disagreements between the two reviewers were resolved with the help of the third author (MDS). 

Data Extraction and Processing

Relevant data from each study were collected using a structured data extraction form through an Excel spreadsheet (Microsoft Corporation, Redmond, Washington, United States), that contained the following items: author, year of publication, journal, included subjects, total study sample size, groups, sample size according to each group, intervention performed, outcome of interest, type of analysis and main findings. The reviewers did not contact any study author once all necessary data was available in the respective studies.

Quality Assessment

The assessment of methodological quality followed a modified version of the Preferred Reporting Items Guidelines for Laboratory Studies in Endodontology (PRILE) and methodology used in previous systematic reviews for in-vitro studies [20,21].

Two reviewers (MDC and PCS) independently assessed the quality of selected studies, and disagreements were discussed to reach a consensus. The parameters considered for the review were: (i) sample size calculation, (ii) information on methods, materials, inputs, samples, specimens, and instruments used in the study that allow methodology replication, (iii) experimental/control groups with similar characteristics, and the method used to ensure similarity of samples, (iv) the process of randomization and allocation concealment, including specifications about random allocation sequence, specimens inclusion, and specimens assignment to the intervention, (v) the process of operator blinding, experiment conduction, and result assessment, (vi) information on data and image management and analysis, including statistics and software, (vii) all the statistical results, including all comparisons between groups, and (vii) details on relevant equipment, software, and settings used to acquire the image(s). 

Each item was judged and classified for each study according to the availability of the information. Classification of "yes" or "no" was attributed to present or absent parameters, respectively, and "some concerns" when the information was not clear. In the end, all studies received one of the following levels: low risk of bias, unclear risk of bias, or high risk of bias. The methodology to determine the overall risk of bias was the following: low risk of bias was assigned when five to eight parameters reported “Yes,” a moderate risk when three to four parameters reported “Yes,” and a high risk of bias was assigned when two or more items reported "Yes".

Data Synthesis

To perform the statistical analysis and meta-analysis, the authors opted for the Stata 17 software (Released 2021; StataCorp LLC, College Station, Texas, United States). In this study, the outcome evaluated was considered a continuous variable. For effect size, Cohen's d test was used, for random effects model, DerSimonian-Laird was used, for heterogeneity, I^2^ (0-40% might not be important, 30-60% may represent moderate heterogeneity, 50-90% may represent substantial heterogeneity, and 75-100% considerable heterogeneity) [19]. p-value <0.05 was considered statistically significant.

Results

Study Selection

**Figure 1:**
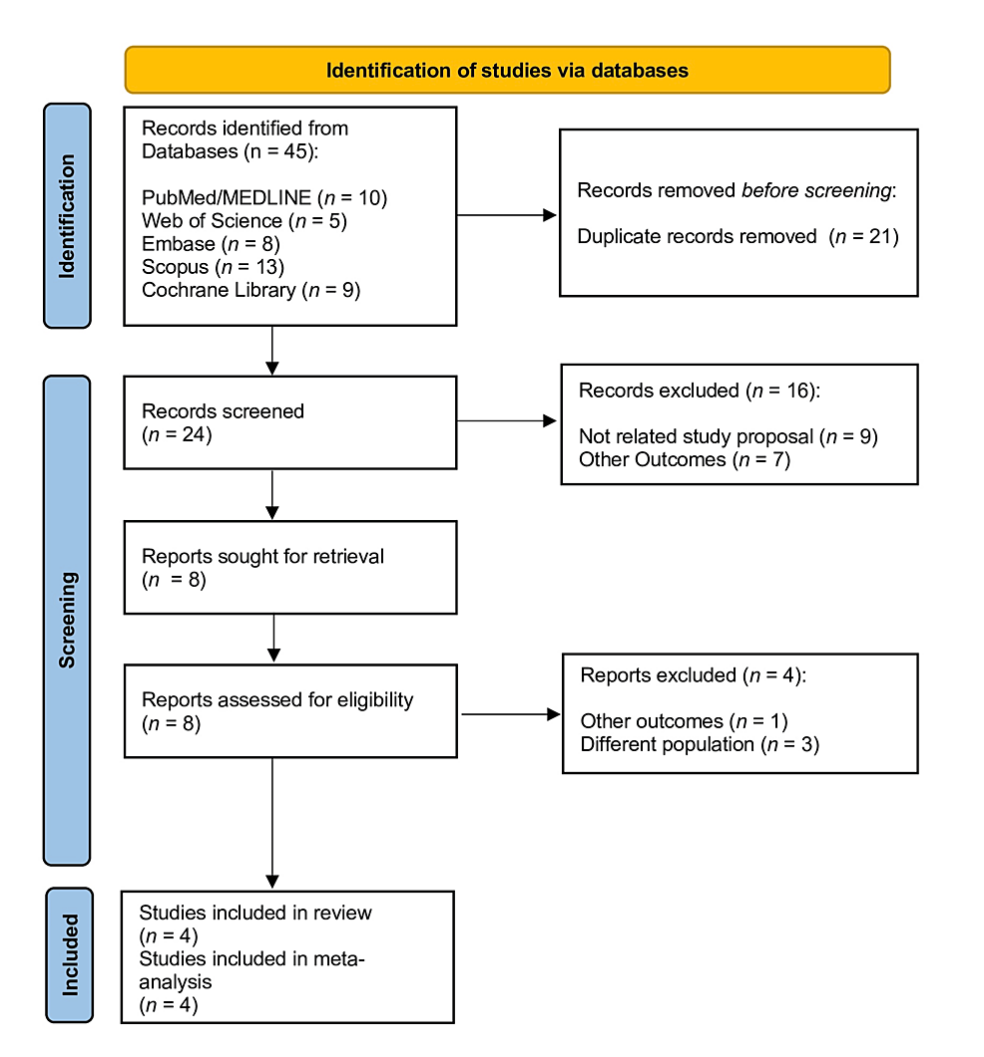
Preferred Reporting Items for Systematic Review and Meta-Analysis (PRISMA) flowchart

Figure 1 represents the study selection according to PRISMA guidelines. After searching the databases, the reviewers found 45 articles; 10 in PubMed/MEDLINE, five in Web of Science, eight in Embase, 13 in Scopus, and nine in the Cochrane Library. Rayyan web application [22] was used to analyze the reference lists of all articles found in the databases and to remove duplicates. Twenty-four studies were selected for title and abstract analysis in the preliminary phase and examined. Sixteen studies were excluded, and eight studies were selected for full-text reading. In this stage, the reviewers excluded four more studies for different reasons; one was excluded as it presented a different outcome than the inclusion criteria, and the remaining three evaluated different samples (not flat or oval root canals). Finally, quantitative and qualitative synthesis included four studies.

Characteristics of the Included Studies

Table 1 describes the main characteristics of the studies. The studies were conducted between 2019 and 2021. Considering the type of tooth used and root canal sample subjects, two studies used oval-shaped canals of permanent mandibular incisors, the third used a flattened distal canal of permanent mandibular molars, and the fourth flattened maxillary second premolars. 

**Table 1:** Characteristics of the included studies Manufacturer details: RECIPROC blue/RECIPROC, VDW Dental, München, Germany; Flatsonic/Clearsonic, Helse Corp., Ocoee, Florida, United States; ProDesign Logic, Bassi Endo, Florida, United States; HyFlex EDM, Coltene Holding, Altstätten, Switzerland

Author	Year	Sample Size	(n)	Groups	Outcome	Main findings
De-Deus et al. [23]	2019	Permanent mandibular Incisors with oval-shaped canal	20	G1: RECIPROC R25 e R40 + RECIPROC 40 G2: RECIPROC R25 e R40 + Clearsonic	Reduction in percentage of non-instrumented walls	Both protocols reduced the percentage of non-instrumented walls; Clearsonic had better results as a supplementary protocol
Rivera-Peña et al. [24]	2019	Permanent mandibular incisors with oval-shaped canal	30	G1: ProDesign Logic 25.05 + PDL 40.05 G2: ProDesign Logic 25.05 + Flatsonic + Clearsonic + ProDesign Logic 40.01	Percentage of non-instrumented surfaces	The group with combined ultrasonic tip and mechanized instrumentation showed a smaller percentage of non-instrumented surfaces
Santos Jr et al. [25]	2020	Flattened Distal Canals of Permanent Mandibular Molars	24	G1: RECIPROC blue (40.06) + Flatsonic G2: ProDesign Logic (40.01 + 40.05) + Flatsonic	Percentage reduction of non-instrumented walls after ultrasonic tip	Flatsonic reduced the percentage of non-instrumented surface after PDL protocol in all canal thirds. RB protocol only reduced the percentage of non-instrumented surfaces in cervical and medium thirds
Tavares et al. [26]	2021	Flattened Maxillary Second Premolars	32	G1: ProDesign Logic (30.01 + 30.05) + Flatsonic + PDL 25.03 G2: HyFlex EDM (10.05 + 25.08) + Flatsonic + Pro Design Logic 25.03	Percentage reduction in non-instrumented surface area after supplementary protocol	Supplementary protocol promoted significant reduction in non-instrumented area after canal preparation, either analyzing surface of different canal thirds and different groups

The mechanized instruments for canal preparation used were RECIPROC (VDW Dental, München, Germany), RECIPROC blue (VDW Dental), ProDesign Logic (Bassi Endo, Florida, United States), and HyFlex EDM (Coltene Holding, Altstätten, Switzerland). Moreover, concerning the canal preparation protocols, one study used reciprocating kinematics, another used the association of rotary and reciprocating, and the last two used the rotary system for canal instrumentation. The ultrasonic tips used to perform the complementary preparation varied between the Flatsonic™ and the Clearsonic™ (Helse Corp., Ocoee, Florida, United States) [23-26]. No studies declared conflicts of interest.

Risk of Bias Assessment

**Figure 2:**
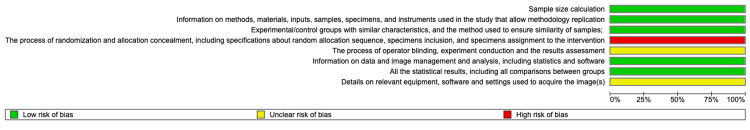
The risk of bias graph

**Figure 3:**
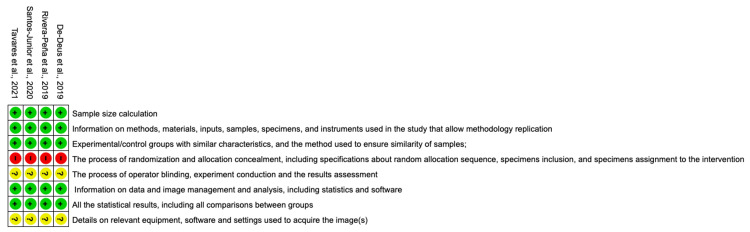
Risk of bias summary References: [23-26]

Figures *2, 3* demonstrate the risk of bias assessment of the included studies. All studies showed a low risk of bias for sample size calculation, information on methods, materials, inputs, samples, specimens, and instruments used in the study that allow methodology replication, experimental/control groups with similar characteristics and the method used to ensure similarity of the sample, information on data management and analysis including testing statistics and software used, and all the statistical results including all comparisons between groups. Regarding the process of blinding the operator who conducted the experiment and the results examiners, details of the equipment, software, and relevant settings used to acquire the image(s), all four studies did not clarify such processes. Randomization and allocation concealment (information on random allocation sequence, selection of specimens, inclusion, and determination of specimen intervention) presented "high risk of bias" for all studies. Regarding the overall risk of bias, the studies showed a low risk of bias.

Meta-analysis

The four studies selected reported quantitative results, allowing the conduction of a quantitative analysis [23-26]. The ultrasonic tip as a supplementary protocol to the preparation presented better results in reduction of the percentage of non-instrumented surfaces with statistical significance [23]. The second study included in this systematic review also demonstrated a smaller percentage of non-instrumented surfaces in the total portion of the root canal (ultrasonic tip) compared to the control group. However, there was no statistical significance [24]. The third study demonstrates that association of mechanized instrumentation in the root canal with ultrasonic tip usage reduced the percentage of non-instrumented surfaces after canal preparation. The rotary instrument shows a reduction in all thirds, and the reciprocating instrument demonstrate reduction of non-instrument surfaces in cervical and middle thirds [25]. The fourth study demonstrated a significant decrease in the non-instrumented surface area after the preparation of the rotary systems in all thirds with statistical significance [26].

**Figure 4:**
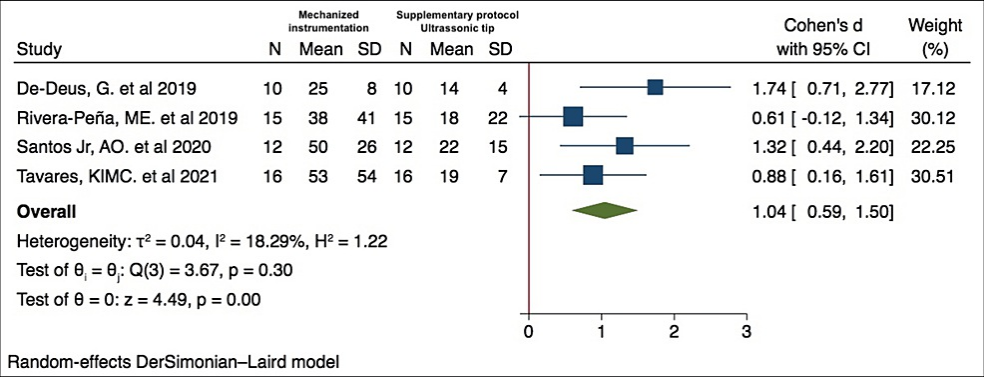
Forest plot comparing the reduction in the percentage of non-instrumented surfaces between the group that performed only mechanized instrumentation and the group that performed supplementary protocol with ultrasonic tip. References: [23-26]

Meta-analysis results showed that the use of the ultrasonic tip after root canal preparation reduced the percentage of non-instrumented surfaces of oval or flattened root canals when compared to the group that performed only mechanized instrumentation, being statistically significant (p< 0.01), as shown in Figure 4. As a result of the heterogeneity test, the I^2^ = 18.29%, confirming a low heterogeneity (I^2^ < 50%). Regarding the effect measures of continuous variables, Cohen's d resulted in a large effect size (Cohen's d: 1.04, 95% CI: 0.59 to 1.50).

Discussion

This systematic review and meta-analysis used data from four in vitro studies analyzed by micro-CT to assess whether ultrasonic tip use reduces the percentage of non-instrumented areas after root canal preparation in an oval or flat root canal. The meta-analysis carried out in this study demonstrated that ultrasonic tip use associated with the mechanized instrument was superior when compared to the group that used only mechanized instrumentation in reducing the percentage of non-instrumented surfaces, with statistical significance (p< 0.01).

The studies concluded that the groups in which they used the ultrasonic tip as supplementary debridement had a reduction in the percentage of non-instrumented areas and improved the cleaning of oval or flat root canals. This result may be associated with the characteristics of the ultrasonic tip, providing better control towards the flattened areas when compared to mechanized preparation instruments, as it has an arrowhead design. In addition, the action obtained by ultrasonic vibration provides a better cleaning action of the root canal [23-26].

Regarding the types of ultrasonic tips used to perform the complementary preparation, Clearsonic and Flatsonic were chosen [23-26]. The Clearsonic tip has a round arrow shape design with a diameter of 0.5 mm, different from the flat arrow design of the Flatsonic tip, which has a diameter of 0.25 mm, providing access to areas that are more difficult to reach. Despite the difference in diameter that may favor the use of Flatsonic in more flat areas, both demonstrated a similar performance. Regardless of the ultrasonic tip used, the physical action of the ultrasonic tip use, in all studies included in our meta-analysis, promoted an increase in dentin removal of the flattened areas where NiTi instruments often cannot reach [23-26]. Furthermore, ultrasonic tip use after NiTi instruments generated a path that facilitates and increases debridement efficiency, providing better reach and sliding of endodontic instruments in difficult access areas [24,26].

Regarding root canal configuration, all studies selected teeth with a transverse configuration two to four times greater than the minimum diameter [23-26]. Wu et al. demonstrate that canals might show different degrees of flatness [10]. According to the literature classification, canals with a buccolingual diameter less than twice the mesiodistal diameter are considered "round or slightly oval". Canals with a buccolingual diameter two to four times greater than the mesiodistal diameter are defined as "long oval or flattened canals", which may vary along the thirds of the tooth [10,27]. The instrumentation of these oval or flattened canals is an anatomical challenge since they have flattened areas that are difficult to access, and difficult in modeling and instrument choice [16]. Previous studies have used different NiTi systems to observe cleaning effectiveness since the morphology of oval or flattened canals does not allow instruments to touch all root canal surfaces [28-30].

In the studies selected for this systematic review and meta-analysis, some authors instrumented the canal with reciprocating and others with rotary instruments, and one study used both systems [23-26]. To verify whether canal preparation could influence the effect of the ultrasonic tip in oval or flattened areas, Santos Jr et al. assessed whether there was a difference by comparing the use of rotary or reciprocating instruments before preparation with the ultrasonic tip and observed that the ultrasonic tip use significantly reduced the percentage of non-instrumented surfaces after using both kinematics [25]. The authors observed that in the ProDesign Logic group, the Flatsonic reduced the percentage of non-instrumented surfaces in all thirds. On the other hand, in the RECIPROC blue group, the ultrasonic tip did not significantly reduce the percentage of non-instrumented surfaces in the apical third. The literature suggests that the association of more than one endodontic instrument, or endodontic instruments with smaller diameters, may promote higher contact with the flattened areas during the brushing movement, improving the effectiveness of the ultrasonic tip [7,25,31]. In the study by Tavares et al., two different rotary NiTi instruments were used (ProDesign Logic and HyFlex EDM). Both preparations provided good access for the use of the Flatsonic, presenting a small difference between them in reducing the number of untouched walls [26]. In the same way, previous studies have analyzed performance in modeling ability by comparing different systems and found similar results, showing unlikely small differences that do not implicate clinical significance [8,32-34]. Furthermore, factors other than kinematics can impact canal cleaning, such as the number of instruments, instrument design, thermal and surface treatment of instruments, and canal anatomy [4,7].

Despite limitations, which are present and inherent in every analysis tool, micro-CT imaging technology is considered an accurate tool to investigate the modeling capability of instruments, comparing root canal morphology before and after preparation in extracted teeth, since it is a non-destructive method and allows a 3D evaluation of the root canal [9,11]. In addition, it is a way to standardize the sample because it guarantees the comparability of the root canal morphology between the experimental groups in the initial phase of the study. One relevant parameter in micro-CT analysis is the amount of unprepared canal surface. Previous studies have revealed that approximately 10-50% of the total canal area remains untouched by instruments, and in oval or flattened canals, the non-instrumented surface ranges from 10% to 80% [2,35-38].

The I^2^ tests showed low heterogeneity (I^2^ < 50%). The studies included in this review compared different root canal preparation techniques and nonidentical instruments, although the outcomes were the same. Sample subjects ranged from oval or flattened permanent mandibular incisors, flattened distal canals of permanent mandibular molars, and flattened permanent maxillary premolars [23-26]. Despite the different dental groups used, the methodology sections of all studies were clear and ensured sample similarity between groups. According to the PRILE guideline, the uniqueness of the root canal anatomy could impact the results of in vitro studies. On the other hand, the anatomical correspondence of the samples allowed groups with similar baseline characteristics, reducing the chance of bias [20]. Such elements are relevant to external validity, allowing extension to other dental groups.

In all studies, the risk of bias of randomization and allocation concealment process was not well described (random allocation sequence, samples included, assignment of specimens intervention). Therefore, all studies had a high risk of bias assessment in this category. Regarding the process of blinding the operator who conducted the experiment and the examiners when assessing the results, the four studies also did not give consistent information about the blinding of the evaluators. According to PRILE, the method of randomization and concealment, as well as sample concealment and allocation, is often not reported or implemented. It is possible to achieve more reliable results when observing these two parameters. However, it is also necessary to note that randomization of samples may not be mandatory in experiments where samples are homogeneous [20].

Based on the findings, all canal preparation techniques leave uninstrumented areas related to complex anatomy that do not match the shape of the preparation instruments. However, a protocol that combines an ultrasonic tip to complement the preparation of difficult-to-reach areas can generate better results, increasing the chances of successful endodontic treatment. To obtain stronger and more conclusive evidence, there is a clear need for more high-quality studies, improving the quality of the information produced.

## Conclusions

This systematic review and meta-analysis demonstrated that ultrasonic tip insert associated with the standard mechanized canal instrumentation shows a reduction in non-instrumented surfaces of oval or flat canal walls, if compared to mechanized canal instrumentation alone. There was a low number of studies meeting the criteria for meta-analysis inclusion, limiting broader analysis and conclusions. Further future well-designed studies must be performed to obtain more conclusive answers.
